# Modelling and predicting the spatio-temporal spread of COVID-19, associated deaths and impact of key risk factors in England

**DOI:** 10.1038/s41598-021-83780-2

**Published:** 2021-03-08

**Authors:** B. Sartorius, A. B. Lawson, R. L. Pullan

**Affiliations:** 1 Department of Disease Control, Faculty of Tropical and Infectious Diseases, London School of Hygiene and Tropical Medicine, Keppel Street, Bloomsbury, London, WC1E 7HT UK; 2 Centre for Tropical Medicine and Global Health, Nuffield Department of Medicine, University of Oxford, Oxford, UK; 3 Department of Health Metrics Sciences, School of Medicine, University of Washington, Seattle, WA USA; 4 Department of Public Health Sciences, Medical University of South Carolina, Charleston, SC 29425 USA

**Keywords:** Epidemiology, Statistics, Viral infection

## Abstract

COVID-19 caseloads in England have passed through a first peak, and at the time of this analysis appeared to be gradually increasing, potentially signalling the emergence of a second wave. To ensure continued response to the epidemic is most effective, it is imperative to better understand both retrospectively and prospectively the geographical evolution of COVID-19 caseloads and deaths at small-area resolution, identify localised areas in space–time at significantly higher risk, quantify the impact of changes in localised population mobility (or movement) on caseloads, identify localised risk factors for increased mortality and project the likely course of the epidemic at high spatial resolution in coming weeks. We applied a Bayesian hierarchical space–time SEIR model to assess the spatiotemporal variability of COVID-19 caseloads (transmission) and deaths at small-area scale in England [Middle Layer Super Output Area (MSOA), 6791 units] and by week (using observed data from week 5 to 34 of 2020), including key determinants, the modelled transmission dynamics and spatial–temporal random effects. We also estimate the number of cases and deaths at small-area resolution with uncertainty projected forward in time by MSOA (up to week 51 of 2020), the impact mobility reductions (and subsequent easing) have had on COVID-19 caseloads and quantify the impact of key socio-demographic risk factors on COVID-19 related mortality risk by MSOA. Reductions in population mobility during the course of the first lockdown had a significant impact on the reduction of COVID-19 caseloads across England, however local authorities have had a varied rate of reduction in population movement which our model suggest has substantially impacted the geographic heterogeneity in caseloads at small-area scale. The steady gain in population mobility, observed from late April, appears to have contributed to a slowdown in caseload reductions towards late June and subsequent start of the second wave. MSOA with higher proportions of elderly (70+ years of age) and elderly living in deprivation, both with very distinct geographic distributions, have a significantly elevated COVID-19 mortality rates. While non-pharmaceutical interventions (that is, reductions in population mobility and social distancing) had a profound impact on the trajectory of the first wave of the COVID-19 outbreak in England, increased population mobility appears to have significantly contributed to the second wave. A number of contiguous small-areas appear to be at a significant elevated risk of high COVID-19 transmission, many of which are also at increased risk for higher mortality rates. A geographically staggered re-introduction of intensified social distancing measures is advised and limited cross MSOA movement if the magnitude and geographic extent of the second wave is to be reduced.

## Introduction

The first cases of COVID-19 in the United Kingdom were confirmed on 31 January 2020^1^.

Despite implementation of non-pharmaceutical interventions, including closure of non-essential services and subsequent and stay at home orders and school closures on 20 and 23 March 2020 respectively, this was not sufficient to contain the first wave of the UK outbreak, although these measures did serve to slow early transmission^2^. The full extent of the effect of the mobility restrictions on COVID-19 transmission has not been fully elucidated nor explicitly quantified^3,4^. In addition, the attributability of hypothesised socio-demographic risk factors for COVID-19 related mortality such as deprivation, crowding and ethnicity have also not been fully quantified nor unpacked at small-area resolution.

As COVID-19 caseloads in England appeared to be rising towards a second peak (wave) at the time of this analysis, to ensure continued response to the epidemic is most effective, it is imperative that we better understand, both retrospectively and prospectively, the geographical evolution of COVID-19 and localised areas in space–time at higher risk of severe disease burden and mortality. Assessing the impact of mobility and population-density (crowding) on differences in caseloads at small area scale will not only allow assessment of the impact that social distance measures have had on the magnitude and timing of the waves, but also allow counterfactual assessment of what this magnitude may have been in the absence of these non-pharmaceutical interventions. More importantly, with the emergence of a subsequent wave(s) and coupled increased likelihood of small-area localised outbreaks, this work could help identify small-areas at elevated risk of transmission and mortality and subsequently inform when it might be safe to start lifting social distancing measures at small-area scale in a geographic staggered approach. Furthermore, a better understanding of the spatiotemporal dynamics of this COVID-19 outbreak, and accurate characterisation of the likely spread and magnitude, will be critical for the design of timely and cost-effective control strategies to minimise the spread of future such pandemics, and to help establish better early warning systems^5–7^.

We apply a Bayesian hierarchical space–time Susceptible-Exposed-Infected-Removed (SEIR) model, previously applied to modelling of the spatial–temporal dynamics of influenza season outbreaks^8^, to publicly available COVID-19 confirmed case and death data in England at small area scale and by week to map the likely trajectory, peak and duration of the outbreak at MSOA level by week, quantifying the impact of mobility. This framework is an extension of the basic model^9^ by combining the SEIR implementation with a spatial conditional autoregressive (CAR) model, and also extend this implementation by considering neighbourhood infection effects for the infection process^8,10^. We utilise weekly Clinical Commission Group (CCG) level population mobility data against observed confirmed COVID-19 case data to assess the impact mobility reduction at small-area scale has had on case transmission, and counterfactually what the magnitude may have been under the scenario of no mobility loss. Furthermore, the inclusion of the death compartment of the model using observed weekly COVID-19 related deaths at MSOA level and linkage to key socio-demographic risk factors allows this framework to identify not only small-areas with significantly elevated mortality risk but also what localised contextual factors may be significantly contributing to this. The space–time dynamics of COVID-19 related mortality in England and associated risk factors/determinants at MSOA level are also assessed to identify vulnerable MSOA.

## Results

### Observed spatial patterns for COVID-19 caseloads at MSOA

**Figure 1:**
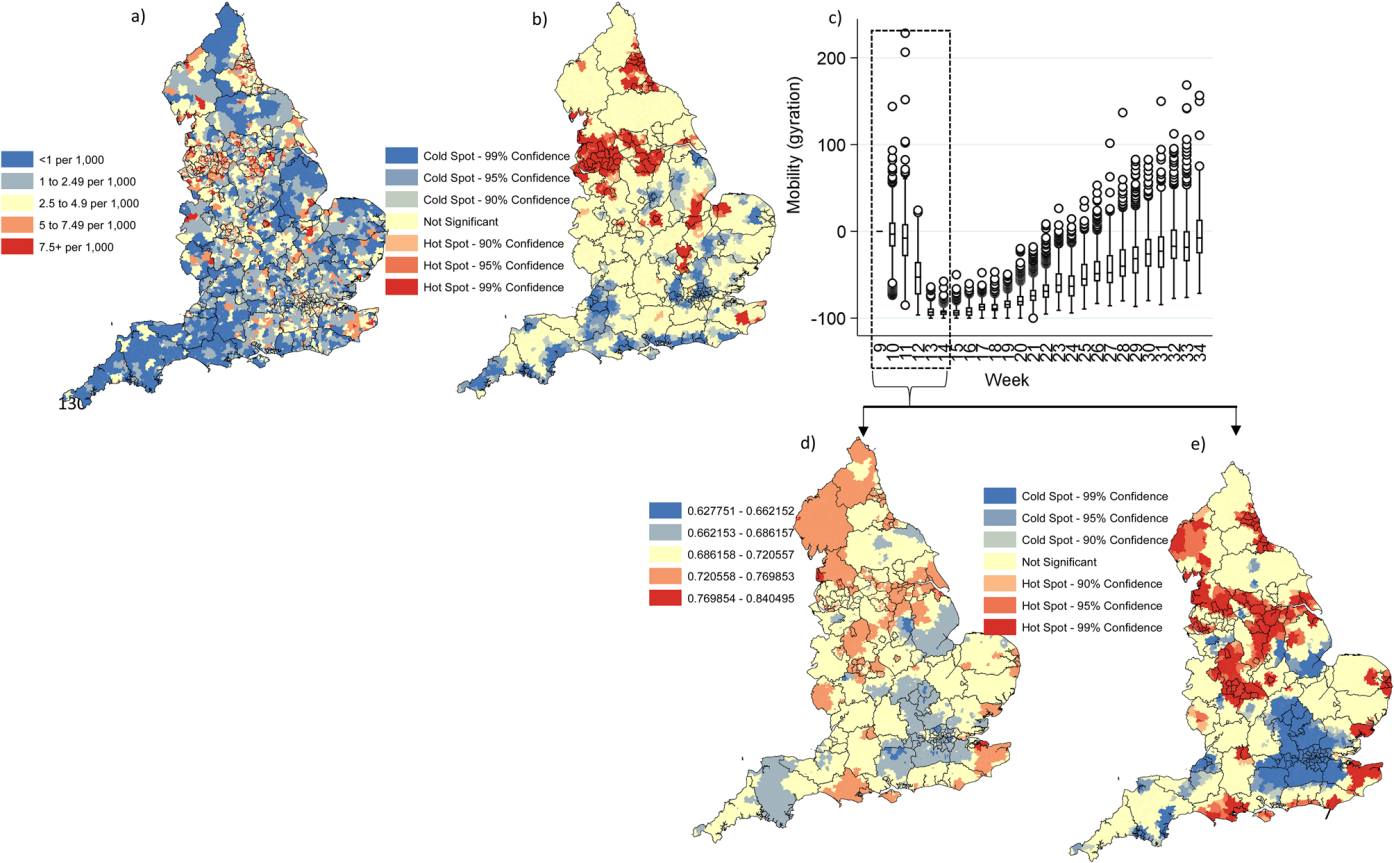
(**a**) cumulative case rates at week 34 at English MSOA level (N = 6791); (**b**) statistically significant localised hot and cold spots for higher and lower cumulative COVID-19 cases at MSOA resolution at week 34 of 2020 using the Getis-Ord Gi* localised clustering statistic; (**c**) Box plot of mobility by MSOA and week; (**d**) cumulative mobility loss (darker red indicates lower mobility loss) by MSOA in England (n = 6791) from week 9 to 14 of 2020; (**e**) statistically significant localised hot and cold spots for higher and lower cumulative mobility loss at MSOA resolution from week 9 to 14 of 2020 using the Getis-Ord Gi* statistic. Maps constructed using ArcGIS 10.5 (https://desktop.arcgis.com/en/).

A spatial assessment of COVID-19 caseloads (scaled by population totals) for the observed data input period (week 9 to 34 of 2020), suggests a non-random distribution to high incidence MSOA across England with higher risk areas concentrated in and around metropolitan areas (Fig. 1a) for example Leicester, Birmingham, Liverpool, and Manchester. The analysis of localised spatial clustering identified significant hot spots (at MSOA level) in Bedford/Bedfordshire, Leicester, Peterborough, large contiguous band in Cheshire and Merseyside, another contiguous band spanning Leeds-Wakefield-Barnsley-Sheffield and in the north Newcastle-Sunderland (Fig. 1b). Additional pockets of significant excess COVID-19 case rates in less metropolitan type areas were identified in pockets throughout England, for example Ashford, Kings Lynn and West Norfolk and Burrow-in-Furness.

### Spatial temporal patterns in population mobility at MSOA

Despite an overall significant reduction in population mobility from early March (week 9) to mid-April (week 14) of 2020 (Fig. 1c), mobility rates began to increase from week 16 and median mobility levels across MSOA had been almost attained pre-lock March/April down levels by week 34 of 2020. The rate of cumulative mobility loss by mid-April varied across MSOA (Fig. 1d), with lower levels of mobility loss generally in part of West and East Midlands, Northwest, York and the Humber land as well as North East England. A large band of significant hotspots for lower cumulative mobility loss at week 14 were identified in a large contiguous aggregation of MSOA spanning from Birmingham through to Leicester, Nottingham, Sheffield, Leeds, Manchester and Liverpool (Fig. 1e). Furthermore, significant clustering of lower cumulative mobility loss was also observed in Newcastle and surrounds, Norwich and coastline areas immediately to the east of Norwich, Tendering/Colchester, Canterbury/Dover and west of Southampton for example.

### Spatial–temporal patterns for COVID-19 mortality

**Figure 2:**
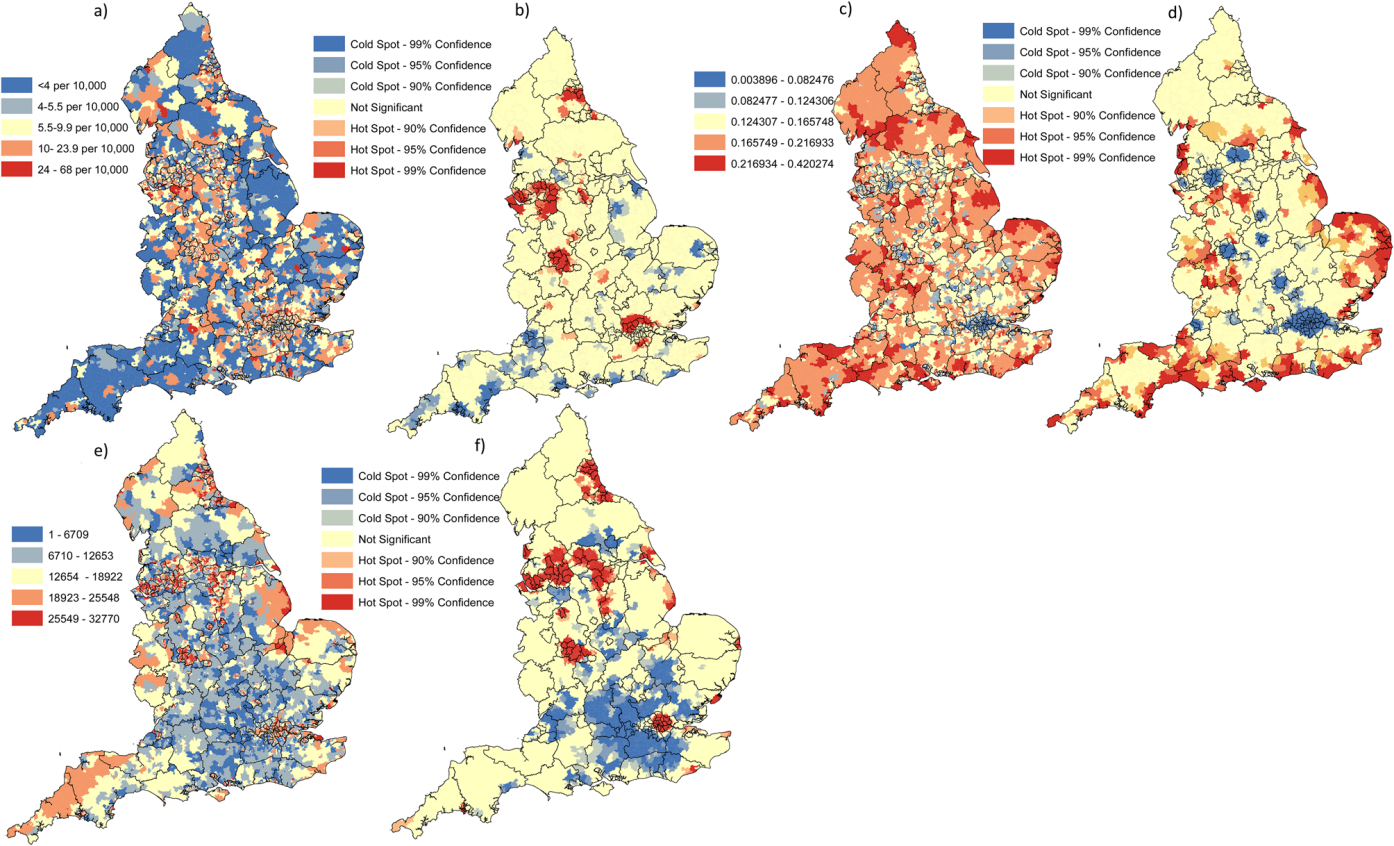
(**a**) Cumulative case rates at week 34 at English MSOA level (N = 6791); (**b**) statistically significant localised hot and cold spots for higher and lower cumulative COVID-19 cases at MSOA resolution at week 34 of 2020 using the Getis-Ord Gi* statistic; (**c**) proportion of population aged 70 + at MSOA resolution; (**d**) statistically significant localised hot and cold spots for higher and lower proportion of population aged 70 + at MSOA resolution using the Getis-Ord Gi* statistic; (**e**) Index of multiple deprivation at MSOA resolution; (**f**) statistically significant localised hot and cold spots for higher and lower proportion of IMD at MSOA resolution using the Getis-Ord Gi* statistic. Maps constructed using ArcGIS 10.5 (https://desktop.arcgis.com/en/).

The highest cumulative death rates for COVID-19 at MSOA resolution from week 9 to 26 of 2020 were observed in parts of North London, most of Birmingham and immediate surrounding MSOA, a large area spanning Cheshire-Merseyside, and lastly in and surrounding Newcastle (Fig. 2a). Significant hotspots for COVID-19 related mortality were highly clustered and were identified in contiguous MSOA located in North London (Ealing-Brent-Hillingdon-Harrow-Barnet-Enfield-Three Rivers-Hertsmere), West Midlands (Birmingham-Sandwell-Walsall-Dudley-Wolverhampton), North West (Liverpool-Manchester-Cheshire), Sheffield, and Newcastle (County Durham-Sunderland-Gateshead) (Fig. 2b). The two principle risk factors associated with COVID-19 mortality risk and leveraged in this study, namely proportion of MSOA population aged 70 years or older and elderly population living in deprivation have two very distinct spatial distributions across England (Fig. 2c,e respectively). The MSOA with higher proportions of population aged 70 years or older appear significantly clustered along the East of England coastline and the coastline in Southwest and South East regions of England (Fig. 2d). Additional significant hot spots for elderly population clustering were identified outside key metropolitan centres, for example: west of Birmingham, north of Liverpool, south of Manchester, north of Nottingham and north of Bradford-Leeds (Fig. 2d).

### Spatial patterns for deprivation

The most deprived (worst 10 percentile) neighbourhoods (680 MSOA) are not evenly distributed across England (Fig. 2e,f), with 50% of these areas located within just 20 local authority districts, notably—Birmingham with 62 such neighbourhoods; Liverpool, 35; Leeds and Manchester, 29 each, and Bradford, 20. Significant spatial clustering of the most deprived neighbourhoods is concentrated in large areas of contiguous MSOA located in London, West Midlands, Northwest, Yorkshire and Northeast England, with pockets of significant deprivation surrounded by less/least deprived neighbourhoods (Fig. 2f). Furthermore, concentrations of deprivation among these 680 MSOA are also disproportionately concentrated in large urban conurbations (142 Industrial and Multi-ethnic, 125 Larger Towns and Cities; 80 Ethnically Diverse Metropolitan Living; 33 urban living), as well as in areas that are (or historically were) classified as heavy industry manufacturing and/or mining sectors (74 manufacturing legacy, 42 service economy; 39 mining legacy). A statistical assessment of spatial clustering for elderly population living in deprivation confirms the significant concentration of hot spots in the metropolitans, namely: London, Birmingham, Liverpool, Manchester, Nottingham, Sheffield-Leeds and Newcastle (Fig. 2f).

### Space–time SEIR dynamics

The full multivariate Bayesian space–time model estimated an asymptomatic fraction of 0.2 (95% UI: 0.06–0.39) i.e. for every 5 confirmed cases there is likely to be 1 additional asymptomatic case (Table 2). A decrease in mobility at MSOA scale has had a significant reduction on caseloads i.e. each unit increase in cumulative mobility (rescaled to be between 0 and 1) appears to be associated with a + 3.6-unit increase in cases on the log scale (95%UI: 3.57–3.61) (Table 1). The change in cumulative mobility also appears to significantly interact with the natural logarithm of population density within a MSOA i.e. the impact of higher mobility is amplified by increasing population density (β =  + 0.05). Increasingly proportion of a MSOA population aged 70 years or older was strongly and significantly associated with increased COVID-19 related deaths (β =  + 4.86, 95% CI: 4.59–5.15) while a decreasing proportion of the elderly population in a given MSOA living in deprivation appears to have a strong and significant negative association (β = − 0.25, 95% CI: − 0.34, − 0.10) (Table 1).

**Table 1:** Posterior statistics from the Bayesian multivariable space–time SEIR model using WinBUGS.

Compartment	Node	Coef. (β)	2.50%	97.50%
Cases	Asymptomatic fraction	+ 0.20	+ 0.06	+ 0.39
	Cumulative mobility loss	+ 3.59	+ 3.57	+ 3.61
	Cumulative mobility loss X ln (Population per km^2^)	+ 0.05	+ 0.05	+ 0.05
	τ (v_1i_)—spatially structured random effect	2.35	2.05	2.70
	τ (U_1i_)—unstructured random effect	14.53	12.06	17.47
Deaths	Proportion aged 70 years and older	+ 4.86	+ 4.59	+ 5.15
	Elderly population living in material deprivation index ^i^	− 0.25	− 0.34	− 0.10
	τ (v_2i_)—spatially structured random effect	0.81	0.73	0.88
	τ (U2_i_)—unstructured random effect	1.44	1.34	1.55

i: increasing score indicate lower level of material deprivation.

### Posterior space–time projections from ST-SEIR model

**Figure 3:**
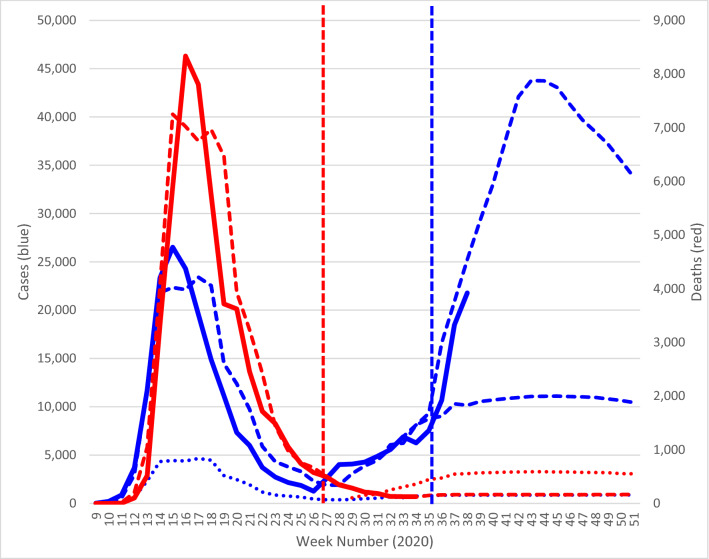
Observed and model fitted number of confirmed cases and deaths in England from week 9 to 34 of 2020, with projections to week 51 of 2020. Vertical coloured line represents the truncation points at the time of this analysis for observed input data for cases (week 34) and deaths (week 26) respectively. Blue solid line = observed number of weekly COVID-19 cases; blue dashed line = model fitted cases; dotted blue line = asymptomatic cases; red solid line = observed number of weekly COVID-19 deaths, red dashed line = model fitted deaths; red dotted line = COVID-19 deaths based on mortality rate from first wave.

The posterior projections from the full space–time SEIR model closely fit with the observed temporal course of case and deaths for COVID-19 over the observed data input period (spanning the first wave), namely week 9 to 26 for deaths and week 9 to 34 for cases (Fig. 3). From week 27 the model fairly closely tracks the increase in caseloads which appears to signal the start of the second wave. Post week 34 i.e. the model out of prediction phase, we note an exponential rise in observed cases beginning around week 35. The model prediction based on averted cases from lockdown in the first phase combined with the impact of steadily increasing population mobility appears to fairly accurately track the exponential rise from week 35 and suggests a likely second wave peak of almost 45,000 weekly cases by week 43/44 (i.e. latter half of October) of 2020 (Fig. 3). Secondly, the model prediction based on the impact of observed mobility change alone, without accounting for the averted (or counterfactual) caseloads in the first wave, appears to significantly underestimate the trajectory of the outbreak in the current phase post week 34 (Fig. 3). Interestingly while the death compartment of the model closely fits the observed death rates in the first wave, the counterfactual projection post week 26 in the death compartment which assumes similar death rates to that observed in the first wave appears to significantly overestimate the death rate observed at the time of this analysis for the initial part of the second wave (Fig. 3).

### Model predicted hotspots for weeks 35–40

**Figure 4:**
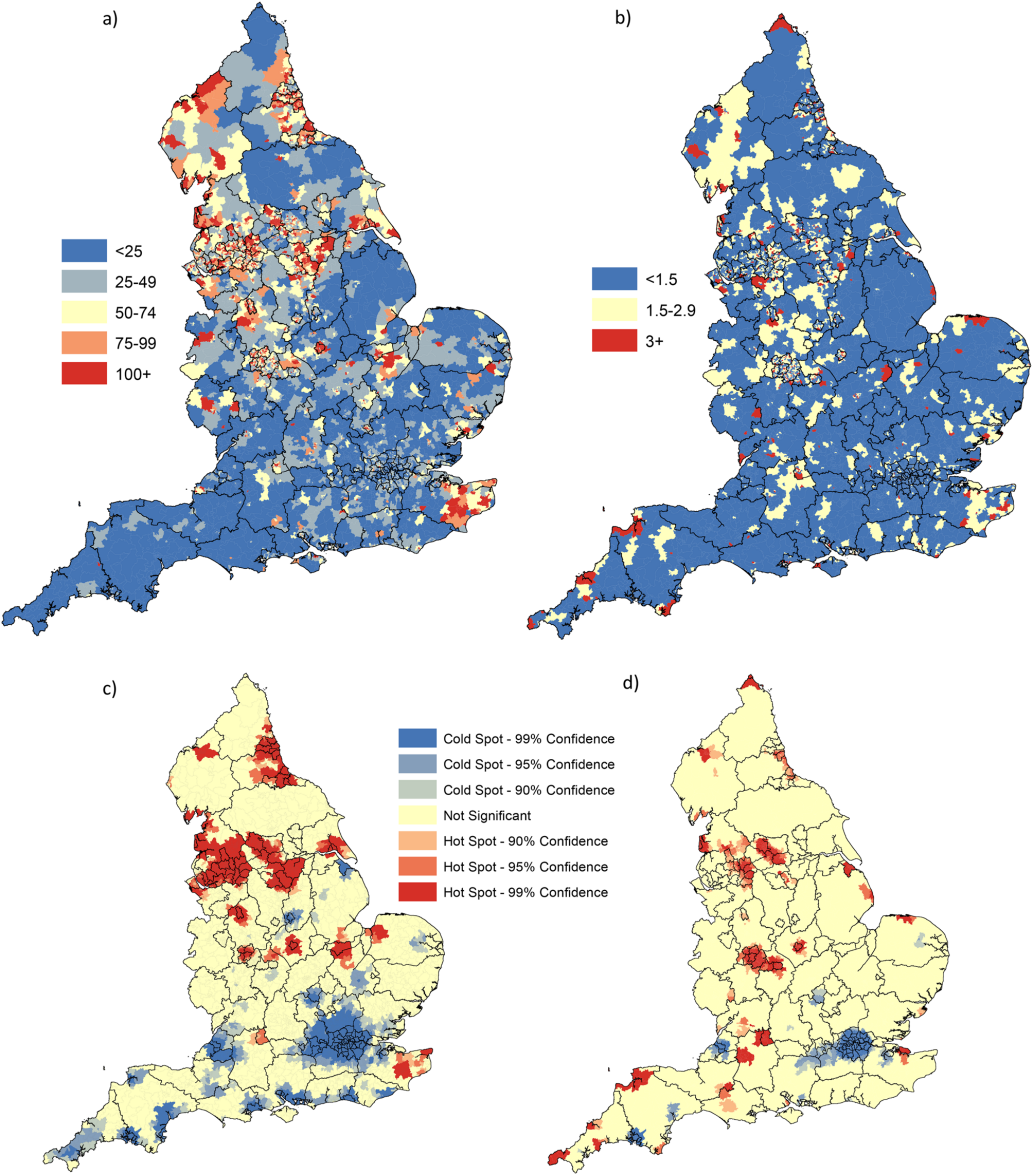
Model projected COVID case (**a**) and death (**b**) rates per 100,000 at MSOA level for week 34 to 40 of 2020; statistically significant localised hot and cold spots for COVID-19 case (**c**) and death (**d**) rates at MSOA level for week 34 to 40, 2020. Maps constructed using ArcGIS 10.5 (https://desktop.arcgis.com/en/).

The forward projection of the model for weeks 35 to 40 at MSOA level suggests high and significant case rates forecast for large contiguous area spanning North West and Yorkshire regions (Liverpool-Manchester complex, contiguous band spanning Bradford-Calderdale-Kirklees-Leeds-Wakefield-Barnsley-Doncaster-Rotherham-Sheffield, contiguous band of MSOA in and around Kingston upon Hull), large band of connected MSOA in the north east spanning from Newcastle through to Stockton-On-Tees, high risk pockets through the midlands (Stoke-on-Trent, Wolverhampton-Walsall-Dudley-Sandwell, Leicester, Peterborough) and two additional pockets in southern England (Swindon, Ashford-Canterbury-Thanet) (Fig. 4a/c). The forecast pockets for significantly high COVID-19 related mortality are more focused/smaller in geographic extent compared to the caseloads with some overlap between areas with project high caseloads and high death rates: Blackpool, Manchester, band spanning Bradford-Leeds-Kirklees-Wakefield, South Tyneside-Sunderland, large band spanning Wolverhampton-Birmingham-Coventry, Leicester and Swindon (Fig. 4b/d). Additional pockets of high mortality rate risk but with low-moderate projected caseload rates were identified, with multiple pockets in the south of England (Wiltshire, North Devon, Cornwall, South Somerset-Dorset and Swale).

## Discussion

To both support and respond effectively to surveillance activities such as NHS Test and Trace or universal weekly testing^11^, it is essential to unpack the spatial–temporal dynamics of the COVID-19 outbreak and related mortality at high spatial resolution (e.g. MSOA) over time and assess the contribution of key risk factors to differences in risk across space and time. Our high resolution spatial–temporal (MSOA by week) model suggest that social distancing measures, as estimated by the impact on population mobility patterns, have had a significant impact on the reduction of COVID-19 caseloads across England, in line with previous studies, for example in China and Italy^12–14^. However, local authorities have seen varied rates of reduction of population movement, significantly impacting the cumulative caseloads at small-area scale. A sensitivity analysis whereby mobility restrictions are eased at various intensities at MSOA scale suggests that a steady gain in mobility, as observed from late April, may have contributed to a slowdown in caseload reductions towards late June and subsequent steady rise thereafter until late August (Fig. 3). Subsequent to that the model prediction and observed caseloads data suggest an exponential rise from week 35 (early September). As such, it would appear that a critical tipping point had been reached at that stage, and our model suggested particular groupings of MSOA at significant increased risk of future caseloads and deaths. Further targeted implementation of local lockdown is essential at this stage. A geographically staggered approach combined with enhanced community surveillance^11^ will become increasingly important if we are to reduce the magnitude of the second wave in England and plan for subsequent waves/cycles in the absence of an effective vaccine.

While previous work^12^ has confirmed that digital sources of mobility data correlate well with COVID-19 incidence and contact mixing^15^ at country level, the extent of the effect of mobility restrictions on COVID-19 transmission in time at small-area resolution has not been fully elucidated nor explicitly quantified. Furthermore, the impact of demographic and socio-economic factors such as elderly population living in deprivation on COVID-19 related mortality risk at high spatial resolution has not been adequately assessed and identification of these areas at excess risk can help guide local policy to better protect these highly vulnerable sub-groups. Lastly, as the Covid-19 pandemic has progressed and evolved, a variety of modelling approaches have been proposed and/or implemented, many of which are time series-based, and do not explicitly incorporate spatial structure in the modelling process. However, it is clear that spatial position (i.e. small area) and influence of neighbouring small-areas is important and so ignoring limits the ability of the models to account for this phenomenon. Additionally, the use of unstructured random effects to allow for unexplained variability and confounding is often ignored. In this article we demonstrate the use of a Bayesian spatial–temporal susceptible-exposed-infected-removed (SEIR) model with spatial geo-referencing at small area (MSOA) resolution in England by week—this combined implementation approach has not been used extensively and presents some distinct advantages (as briefly summarised above) over either formulation in isolation.

Crucially, our results highlight differences in epidemic dynamics across small areas in England, emphasising the importance of monitoring at fine sub-national scale. Our findings suggest that a significant proportion of caseload and death variance is spatially structured, and that caseloads in neighbouring local authorities of a given MSOA in the preceding time point are significantly associated with caseloads in current time point. This—coupled with the large significant regional clustering of high caseloads observed—has important implications for informing limitations on movement between areas in the subsequent phases of lockdown easing. Small-area lockdowns could help reduce COVID-19 transmission while minimizing the size of the population (and economy) that needs to be disrupted to continue to release reduced transmission. Furthermore, deaths due to COVID-19 appear to be significantly clustered in space, with some overlap between areas in and around metropolitan centres with higher levels of elderly population living in deprivation (prominent examples: Wolverhampton-Birmingham complex, Blackpool, Manchester, Bradford-Leeds-Wakefield complex and south of Newcastle). However additional pockets of excess mortality risk were also identified in areas in less deprived coastal areas, however from our analyses many of these localities have much higher proportions of elderly population which may in part explain these clusters.

Human mobility has been demonstrated to play an important role in the early stages of the COVID-19 epidemic^12^ and in particular reductions observed in digital data sources of mobility appear to correlate well with incidence^13^ as well as contact patterns^15^. As many countries continue to bring their COVID-19 outbreaks under control, evidence on the effectiveness of current lockdowns continues to emerge^16–19^. Social distancing measures have had a significant impact on the reduction of COVID-19 caseloads across England, however local authorities have had a varied rate of reduction of population movement, which has substantially impacted the cumulative caseloads at small-area scale. While non-pharmaceutical interventions such as social distancing have had a profound impact on the trajectory of the COVID-19 outbreak in England, country wide relaxing of these measures from June 2020 and gradual observed increases in population mobility which started even prior to this, risks a rebound in a number of local authorities which still have caseloads above the threshold required to realise MSOA cessation.

Furthermore, at the time of this analysis and the period covered by the input data, vaccination was not yet available for SARS-CoV-2. An application of this model post vaccine rollout would need to explicitly account for increasing cumulative vaccination of the population in a given MSOA with time. This could be achieved using a modified version of the SEIR model that accounts for vaccination, for example^20^.

SARS-CoV-2 co-infection with other infectious agents at admission have been documented^21^ and appear to significantly increase mortality risk^22^. Future applications should consider additional potentially useful and important predictors for COVID-19 mortality such as co-infection rates at admission.

This study is subject to limitations. First, confirmed COVID-19 cases were linked to the MSOA where the test was carried out, rather than patient residence. This is of greater concern for metropolitan areas, where a substantial proportion of patients may have been admitted or transferred to neighbouring trusts before being tested. Furthermore, we model the impact of mobility within a given MSOA and week on caseloads and thus do not include a measure of between MSOA mobility by week which would also be an important additional driver of wider COVID-19 related spread and subsequent caseloads. Secondly, the data are also vulnerable to bias introduced by variable testing rates between Trusts, which may have contributed to greater between MSOA variability. Thirdly, we are assessing the association between aggregated cases/deaths at MSOA level with aggregated risk factor variables at the same level. Thus we cannot discount the possible impact of ecological fallacy in the observed association. Fourthly, both the case fatality risk and the fraction of asymptomatic cases of COVID-19 are known to vary widely by age^23,24^. Thus our model does not fully consider the age-structured transmission and mortality dynamics and could this lead to biased estimates due to possible variations in the age-distribution of each MSOA. A final limitation relates to the mobility data and lack of detail regarding movement between local authorities. While we have demonstrated the importance of mobility loss within a given MSOA on its subsequent caseloads, the impact of movement between local authorities (and density thereof) would provide additional important information regarding cross locality mixing and further targeted restriction of movement between particular higher risk areas.

## Conclusions

This study highlights the importance and usefulness of space–time framework to unpack the dynamics of the COVID-19 outbreak at high resolution (MSOA) and in time, identify particular small-areas at elevated risk of transmission as well as COVID-19 related mortality. Furthermore, at small area scale we demonstrated the utility of longitudinal mobility data for real-time surveillance of the impact of social distancing interventions, and thereby can also allow national authorities to assess the impact on outbreak dynamics at small-area. It is advisable to pre-emptively assess what impact changes in mobility post lockdown easing will have on outbreak rebound potential at localised geographic scale, and to include monitoring of changes in daily/weekly mobility patterns. Lastly geographic areas with high proportions of elderly population and in particularly elderly population living in deprivation are at significantly greater risk of COVID-19 related mortality, and therefore enhanced surveillance/case follow-up should be ensured in these more vulnerable areas.

## Methods

### Overview

This analysis adheres to the Guidelines for Accurate and Transparent Health Estimates Reporting standards (GATHER)^25^ (Supplementary Material Section 1). The data used are all in the public domain. Data processing and analyses were performed using Stata 16, and WINBUGS. The statistical code for implementing the Bayesian model in WINBUGS is provided in Supplementary Material (Supplementary Material Section 2). We constructed all maps using ArcGIS 10.5.

### Data

A summary of the input data and sources is presented in Table 2. The weekly number of confirmed COVID-19 cases (Pillar 1—Government's mass testing programme) and deaths for each MSOA Middle Layer Super Output Area (MSOA) (6791 areas) in England were obtained from the UK COVID-19 dashboard (https://coronavirus.data.gov.uk/) for the period of February 24, 2020 (week 5) to August 23, 2020 (week 34). The MSOA shape file, used to create the adjacency matrix for the model below and for mapping of the model posteriors, was obtained from the United Kingdom government geoportal platform: https://geoportal.statistics.gov.uk/datasets/middle-layer-super-output-areas-december-2011-boundaries-ew-bfc-1. Weekly COVID-19 deaths at MSOA resolution were also extracted from the NHS PHE Dashboard (https://coronavirus.data.gov.uk/).

**Table 2:** List of model components, relevant variables and data sources.

Model component	Variable	Source (open access unless indicated*)	Spatial resolution	Temporal resolution	Compartment
Outcomes	Confirmed cases	UK COVID-19 Dashboard: https://coronavirus.data.gov.uk/ Number of people with a lab-confirmed positive COVID-19 PCR test. Data include only pillar 1 cases until 2 July, from when pillar 2 cases are also included. Cases are allocated to the person's area of residence	MSOA	Weekly	Cases
	Deaths within 28 days of positive test result for COVID-19	UK COVID-19 Dashboard: https://coronavirus.data.gov.uk/ Total number of people who had a positive test result for COVID-19 and died within 28 days of the first positive test, reported on or up to the date of death or reporting date (depending on availability). Deaths are allocated to the deceased's usual area of residence	MSOA	Weekly	Deaths
Covariate (daily varying)	Mobility (population movement index)	Oxford COVID-19 impact monitor (Cuebiq) (https://oxford-covid-19.com/)	Clinical Commission Group (CCG)	Daily	Cases
Covariates (annual estimates, weekly fixed)	Age, ethnic structure	ONS (https://www.ons.gov.uk/)	MSOA	2020	Deaths
	Population density	ONS (https://www.ons.gov.uk/)	MSOA	2020	Cases
	Household occupancy (overcrowding)	ONS (https://www.ons.gov.uk/)	MSOA	2019	Cases/deaths
	Index of Multiple Deprivation (IMD)	Multiple deprivation experienced by Lower Layer Super Output Area (LSOA) in England (https://www.gov.uk/government/statistics/english-indices-of-deprivation-2019)	MSOA	2019	Deaths
	Elderly population proportion living in deprivation	Public Health England – Local Health: https://www.localhealth.org.uk/	MSOA	2019	Deaths
	Emergency hospital admissions for chronic disease	ONS (https://www.ons.gov.uk/)	MSOA	2017/18	Deaths

Data on population size, population density and the proportion of the population aged 70 years and older as well as proportion of population that is of Black or mixed ethnicity by MSOA were obtained from the Office for National Statistics (ONS) (https://www.ons.gov.uk/). Furthermore, we also extracted the Index of Multiple Deprivation (IMD) score and proportion of the elderly population living in deprivation for each MSOA in England for 2019 (Public Health England: Local Health. [Accessed: 24/08/2020] https://www.localhealth.org.uk/#c=home).

Daily population movement data by Clinical Commission Group (CCG) was extracted from the COVID-19 Impact monitor (https://www.oxford-covid-19.com/). Weekly mobility in a given MSOA where assumed to be the same as the weekly mobility in the higher level CCG containing most of that MSOA. Furthermore, rail passenger numbers and crowding statistics (2018) were extracted for use a commuter density index by MSOA prior to the local down for use a proxy for likely daily contacts with other individuals and mixing density (http://maps.dft.gov.uk/rail-passengers-and-crowding/interactive-dashboard/index.html.

As a proxy for underlying chronic circulatory and respiratory disease burden, we also utilised mortality rates from chronic obstructive pulmonary disease (COPD) from 2016–2018 by MSOA. (Public Health England: Local Health. [Accessed: 24/08/2020] https://www.localhealth.org.uk/#c=home).

### Data analysis

For initial exploratory spatial analysis, we used the Getis–Ord Gi ∗ statistic (Gi ∗)^26^, also known as hot-spot analysis, to identify significant higher and lower risk MSOA in terms of case and deaths rates for the observed input data period, namely week 9 to 34. The significance level was set as 0.05. A previously developed Bayesian space–time SEIR formulation^8^ was then applied to assess the spatiotemporal variability of COVID-19 transmission at small area scale (MSOA) and by week in England, by accounting for the modelled transmission dynamics of the pathogen, inherent spatial–temporal correlation in the data, and important contextual risk factors for both COVID-19 cases and deaths. We also assessed the sum of cases in shared neighbours in the preceding time point as an additional parameter in our model to further assess the dependence of caseloads in adjoined areas^27^. Based on the available data provided we the starting study week was denoted as week 9 of 2020 (starting 24 February 2020), with available case data at the time of extraction for this analysis available up to week 34 (starting 17 August 2020) and death data available up to week 26 (starting 22June 2020). We also limited the end date for the input data at week 34 to assess the predictive performance of the model into the future if we are to prove its predictive utility and thus use as a pre-emptive resource allocation/prioritization tool.

Let I_ij_ be weekly confirmed COVID-19 cases in MSOA region *i* and week *j*, (*i* = 1*,…,*151*; j* = 9,…, 51). A discrete form SEIR model for the size of susceptible population at week (*j* + 1) and MSOA region *i* is given by S_i,j+1_ = S_i,j_ − I_i,j_-Ri_,j_, where *S*, *I* and R represent the susceptible, infectious and removed (deaths and recovered) populations, respectively. The number of the infected COVID-19 cases in region *i* and week *j* were assumed to follow a Poisson distribution, with expected number of cases is a function of the serial interval or generation time as a time measure of disease communicability and defined as the number of infected cases in one median incubation period back (*j* − 1 week^28^) i.e. and the susceptible population size in week *j.* The number of asymptomatic cases by week and MSOA is assumed to be a function of the observed case load in a given MSOA/week multiplied by a parameter for the additional fraction that are asymptomatic.

Hence, I_ij_ ~ Pois(μ_ij_), and μ_ij_ is given by ^8^:

$$\begin{aligned} &\upmu _{{{\text{ij}}}} =\upbeta _{{{\text{ij}}}} \times {\text{S}}_{{{\text{ij}}}} \times {\text{I}}_{{{\text{i}},{\text{j}} - 1}} \\ & {\text{S}}_{{{\text{ij}}}} = {\text{S}}_{{{\text{ij}} - {1}}} - {\text{I}}_{{{\text{ij}} - {1}}} - {\text{Asym}}_{{{\text{ij }}{-}{ 1}}} - {\text{ deaths}}_{{{\text{ij}} - {1}}} \\ & {\text{log}}\left( {\upmu _{{{\text{ij}}}} } \right) = {\text{log}}\left( {\upbeta_{{{\text{1ij}}}} } \right) + {\text{log}}\left( {{\text{S}}_{{{\text{ij}}}} } \right) + {\text{log}}\left( {{\text{I}}_{{{\text{i}},{\text{j}} - {1}}} } \right) \\ & {\text{log}}\left( {\upbeta _{{{\text{1ij}}}} } \right) = b_{1} 0 + b_{{1}} \times \left( {{\text{mobility}}} \right)_{{{\text{i}},{\text{j}}}} + b_{{2}} \times {\text{mobility}}_{{{\text{i}},{\text{j}}}} \, \times {\text{ln}}\left( {{\text{population density}}_{{\text{i}}} } \right) + {\text{u}}_{{{\text{1i}}}} + {\text{v}}_{{{\text{1i}}}} \\ & {\text{Asym}}_{{{\text{ij}}}} \sim {\text{Pois}}\left( {{\text{U}}_{{{\text{ij}}}} *{\text{asym}}} \right) \\ & {\text{asym}}\sim {\text{Uniform}}\left( {0.0{5},0.{4}} \right) \\ \end{aligned}$$

And deaths_ij_ ~ Pois(dmu_ij_), and dmu_ij_ is dependent on caseloads 2 weeks prior^29^ and given by

$$\begin{aligned} {\text{log}}\left( {{\text{dm}}\upmu _{{{\text{ij}}}} } \right) & = {\text{log}}\left( {\upbeta _{{{\text{2ij}}}} } \right) + {\text{log}}\left( {{\text{I}}_{{{\text{i}},{\text{j}} - {2}}} } \right) \\ {\text{log}}\left( {\upbeta _{{{\text{2ij}}}} } \right) & = b_{2} 0 + b_{{3}} \times \left( {{\text{p70plus}}} \right)_{{\text{i}}} + b_{{4}} \times \left( {{\text{e-IMD}}} \right)_{{\text{i}}} + {\text{u}}_{{{\text{2i}}}} + {\text{v}}_{{{\text{2i}}}} \\ \end{aligned}$$

Here *b* = (*b*_0_, *b*_1_, *b*_2_) is the vector of regression coefficients for the intercept (representing the log-transformed baseline transmission rate across all locations), mobility represents the observed weekly mobility in a given MSOA based on the mobility in the CCG containing most of that MSOA; mobility x population represents an interaction term to capture the varying impact of mobility change as a function of changing population density at MSOA level; p_70plus_ plus represents the proportion of the population in a given MSOA that were 70 years or older in 2020 in a given MSOA; e-IMD represents the proportion of the elderly people living in deprivation in a given MSOA in 2019; *u*_*i*_ corresponds to structured (spatial) heterogeneity and represents spatial variation in transmission rate between regions that captures the effects of unobserved variables with an underlying spatial pattern; *v*_*i*_ correspond to geographically unstructured (i.e. random effect) heterogeneity in the transmission rate; and μ_*ij*_ is the weekly transmission rate at MSOA *i* and week *j* after incorporating the spatiotemporal effects of local social-demographic factors, the transmission dynamics of COVID-19 outbreak (i.e. SEIR model) and random effects (*u*_*i*_ and *v*_*i*_). All covariate coefficients had diffuse normal priors, given by ***b*** ~ *N*(0.0, 1.0E6). The variances of the random effects had uniform priors, *σ*_*u*_ ~ *U*(0,5) and σ_*v*_ ~ *U*(0,5). We assumed that the asymptomatic proportion parameter, asym, was constrained to between 5 to 40%. This is guided by two systematic reviews^30,31^ which suggest that between 17% (95% CI 14–20%) and 20% (95% CI 17–25%) of infections respectively are asymptomatic. However, given that there is still substantial heterogeneity across these studies, we chose a more conservative (wider uncertainty) range for this fraction in our model, hence the distributional assumption of 5 to 40% in our model.

A convolution CAR model^32^ was applied to decompose the log of area-level relative risks into the sum of two random effects, namely a structured spatial dependency effect and unstructured areal level heterogeneity effect through the random effects *u*_*i*_ and *v*_*i*_ respectively. The convolution approach has demonstrated good robust estimates across multiple studies and a range of disease clustering/modelling scenarios, for example^33^.This spatial structured effect models the effect of proximity using a first-order neighbourhood structure (i.e. MSOA adjacency matrix), whereby the random effect is assumed to have a normal distribution, with the conditional weighted mean given by the average of the neighbours.

We conducted various sensitivity analyses to estimate the impact of social distancing (as proxied by the daily population mobility covariate) on COVID-19 caseloads in England. The model coefficients for the mobility covariates from the full multivariable space–time model was applied to the baseline mobility prior to lockdown and propagated throughout the period assuming no change in mobility from baseline (that is, daily mobility patterns remained unchanged). Secondly, the observed rate of change of mobility at week 34 was propagated forward to week 51. Lastly, we assumed various fractions of mobility gain post week 34 and applied these to the difference between the counterfactual of no reduction in population movement and observed first wave outbreak dynamics under full lockdown to characterise the likely prospective trajectory in England in the coming weeks.

A comparison of model fit was performed using the deviance information criterion (DIC). Posterior distributions for parameters of interest were obtained through Markov chain Monte Carlo (MCMC) sampling. Convergence was assessed by visual inspection of the trace/autocorrelation plots for the sample chains, using Gelman–Rubin statistical plots^34^ and by confirming the MCMC error for all posteriors were less than 5% of the standard deviation for a given posterior. We ran 100,000 MCMC iterations and discarded the first 10,000 MCMC iterations as part of the model burn-in. We extracted the mean point estimate for a given posterior as well as the 95% uncertainty intervals (2.5 to 97.5 percentile). This analysis was performed using WinBUGS version 1.4.3.

We validated the performance of the model using out of sample prediction by comparing the predicted number of cases from the model post week 34 (observed case data truncation point) with the observed cases to prove its utility as an early warning (pre-emptive) tool for resource allocation and prioritization by MSOA resolution (Supplementary Material Section 3).

## Supplementary Information

Supplementary Information.
