# Supplementary material for: Modelling and predicting the spatio-temporal spread of COVID-19, associated deaths and impact of key risk factors in England
Source: Sci Rep. 2021 Mar 8;11:5378. doi: 10.1038/s41598-021-83780-2 (PMC7940626; doi:10.1038/s41598-021-83780-2)
Supplement: Supplementary file 1 — Supplementary Information. [file 41598_2021_83780_MOESM1_ESM.docx]

**Supplementary Material**

Modelling and predicting the spatio-temporal spread of COVID-19, associated deaths and impact of key risk factors in England

Sartorius B ^1,2,3^*, Lawson AB ^4^, Pullan RL ^1^

1 Department of Disease Control, Faculty of Tropical and Infectious Diseases, London School of Hygiene & Tropical Medicine, Keppel Street, London, WC1E 7HT, UK

2. Centre for Tropical Medicine and Global Health, Nuffield Department of Medicine, University of Oxford, UK

3. Department of Health Metrics Sciences, School of Medicine, University of Washington, Seattle, WA, USA

4 Department of Public Health Sciences, Medical University of South Carolina, Charleston

29425, South Carolina, USA

*Correspondence: Dr Benn Sartorius; Address: Centre for Tropical Medicine and Global Health, Nuffield Department of Medicine, University of Oxford, New Richards Building, Old Road Campus, Roosevelt Drive, Oxford, OX3 7LG, UK; Email: benn.sartorius@ndm.ox.ac.uk.

**Section 1.** Gather checklist of information that should be included in new reports of global health estimates

| Item # | Checklist item | Reported on page # |
| --- | --- | --- |
| Objectives and funding | | |
| 1 | Define the indicator(s), populations (including age, sex, and geographic entities), and time period(s) for which estimates were made. | 1 |
| 2 | List the funding sources for the work. | 2 |
| Data Inputs | | |
| *For all data inputs from multiple sources that are synthesized as part of the study:* | | |
| 3 | Describe how the data were identified and how the data were accessed. | 3,4,Table 1 |
| 4 | Specify the inclusion and exclusion criteria. Identify all ad-hoc exclusions. | 3 |
| 5 | Provide information on all included data sources and their main characteristics. For each data source used, report reference information or contact name/institution, population represented, data collection method, year(s) of data collection, sex and age range, diagnostic criteria or measurement method, and sample size, as relevant. | 3,4,Table 1 |
| 6 | Identify and describe any categories of input data that have potentially important biases (e.g., based on characteristics listed in item 5). | N/A |
|  | | |
| 7 | Describe and give sources for any other data inputs. | 3,4,Table 1 |
| *For all data inputs:* | | |
| 8 | Provide all data inputs in a file format from which data can be efficiently extracted (e.g., a spreadsheet rather than a PDF), including all relevant meta-data listed in item 5. For any data inputs that cannot be shared because of ethical or legal reasons, such as third-party ownership, provide a contact name or the name of the institution that retains the right to the data. | Table 1 |
| Data analysis | | |
| 9 | Provide a conceptual overview of the data analysis method. A diagram may be helpful. | 3 |
| 10 | Provide a detailed description of all steps of the analysis, including mathematical formulae. This description should cover, as relevant, data cleaning, data pre-processing, data adjustments and weighting of data sources, and mathematical or statistical model(s). | 7-9 |
| 11 | Describe how candidate models were evaluated and how the final model(s) were selected. | 6-8 |
| 12 | Provide the results of an evaluation of model performance, if done, as well as the results of any relevant sensitivity analysis. | Supplementary 3  8 |
| 13 | Describe methods for calculating uncertainty of the estimates. State which sources of uncertainty were, and were not, accounted for in the uncertainty analysis. | 9 |
| 14 | State how analytic or statistical source code used to generate estimates can be accessed. | 5, Supplementary 2 |
| Results and Discussion | | |
| 15 | Provide published estimates in a file format from which data can be efficiently extracted. | Supplementary 5 |
| 16 | Report a quantitative measure of the uncertainty of the estimates (e.g. uncertainty intervals). | 9-13 |
| 17 | Interpret results in light of existing evidence. If updating a previous set of estimates, describe the reasons for changes in estimates. | 13-15 |
| 18 | Discuss limitations of the estimates. Include a discussion of any modelling assumptions or data limitations that affect interpretation of the estimates. | 15 |

**Section 2:** WinBUGS code for full space-time SEIR model

model{

for (i in 1:N){

b1.ut[i]~dnorm(alpha[1],tau.bu[1])

b2.ut[i]~dnorm(alpha[2],tau.bu[2])

deaths_mod[i,1]~dpois(mu.d[i,1])

mu.d[i,1]<-0

susc[i,1]<-Pop_AllAges[i]

cases_mod[i,1]~dpois(mu.c[i,1])

mu.c[i,1]<-0

asym.cases[i,1]<-cases_mod[i,1]*asym

deaths_mod[i,2]~dpois(mu.d[i,2])

mu.d[i,2]<-0

susc[i,2]<-susc[i,1]-cases_mod[i,1]-inf[i,1]-deaths_mod[i,1]

cases_mod[i,2]~dpois(mu.c[i,2])

log(mu.c[i,2])<-b1[i]+b1.ut[i]+beta[1]*cum_gyration[i,2]

asym.cases[i,2]<-cases_mod[i,1]*asym

for (j in 3:T){

deaths_mod[i,j]~dpois(mu.d[i,j])

log(mu.d[i,j])<-b2[i]+b2.ut[i]+log(cases_mod[i,j-1]+0.001)+beta[3]*prop_70plus[i]+beta[4]*imd19_inv[i]

susc[i,j]<-susc[i,j-1]- asym.cases[i,j-1]-cases_mod[i,j-1]-deaths_mod[i,j-1]

cases_mod[i,j]~dpois(mu.c[i,j])

log(mu.c[i,j])<-b1[i]+b1.ut[i]+log(susc[i,j]+0.001)+beta0[1]*log(cases_mod[i,j-1]+0.001)+beta[1]*cum_gyration[i,j-1]+beta[2]*imd19_inv[i]

asym.cases[i,j]<-cases_mod[i,j]*asym

}

}

b1[1:N] ~ car.normal(adj[], weights[], num[], tau.b[1])

b2[1:N] ~ car.normal(adj[], weights[], num[], tau.b[2])

for(k in 1:sumNumNeigh) {

weights[k] <- 1

}

alpha[1]~dflat()

alpha[2]~dflat()

beta0[1]~dunif(0,1)

beta[1]~dnorm(0,1)

beta[2]~dnorm(0,1)

beta[3]~dnorm(0,1)

beta[4]~dnorm(0,1)

for (k in 1:2){

tau.b[k]~dgamma(0.5,0.0005)

}

for (k in 1:2){

tau.bu[k]~dgamma(0.5,0.0005)

}

asym~dunif(0.05,0.4)

for (i in 1:N){

gyration.baseline[i]<-cum_gyration[i,1]

mu.counter[i,1]<-0

mu.counter[i,2]<-mu.c[i,2]

for (j in 3:T){

log(mu.counter[i,j])<-b1[i]+b1.ut[i]+log(susc[i,j]+0.001)+beta0[1]*log(cases_mod[i,j-1]+0.001)+beta[1]*gyration.baseline[i]

}

}

for (j in 1:T){

cases.fitted[j] <- sum(mu.c[,j])

asym.fitted[j] <- sum(asym.cases [,j])

cases.fitted.counter[j] <- sum(mu.counter[,j])

deaths.fitted[j] <- sum(mu.d[,j])

}

}

**Section 3:** Model fit and out of sample validation/performance

The model appears to adequately fit the observed number of cases by MSOA for the period of week 9 to week 34 of 2020 which corresponds to observed data input period for the model (Figure 3.1, Spearman rank correlation coefficient=+0.966, p<0.001. A comparison of model fitted cases for a 20% randomly withheld sample of observed data points for the period of week 9 to 34 against total observed cases by MSOA appears to suggest a high degree of correlation (Spearman rank correlation coefficient = +0.867, p<0.001) (Figure 3.2). Thus we believe that the model has demonstrated good out of sample predictive capabilities.


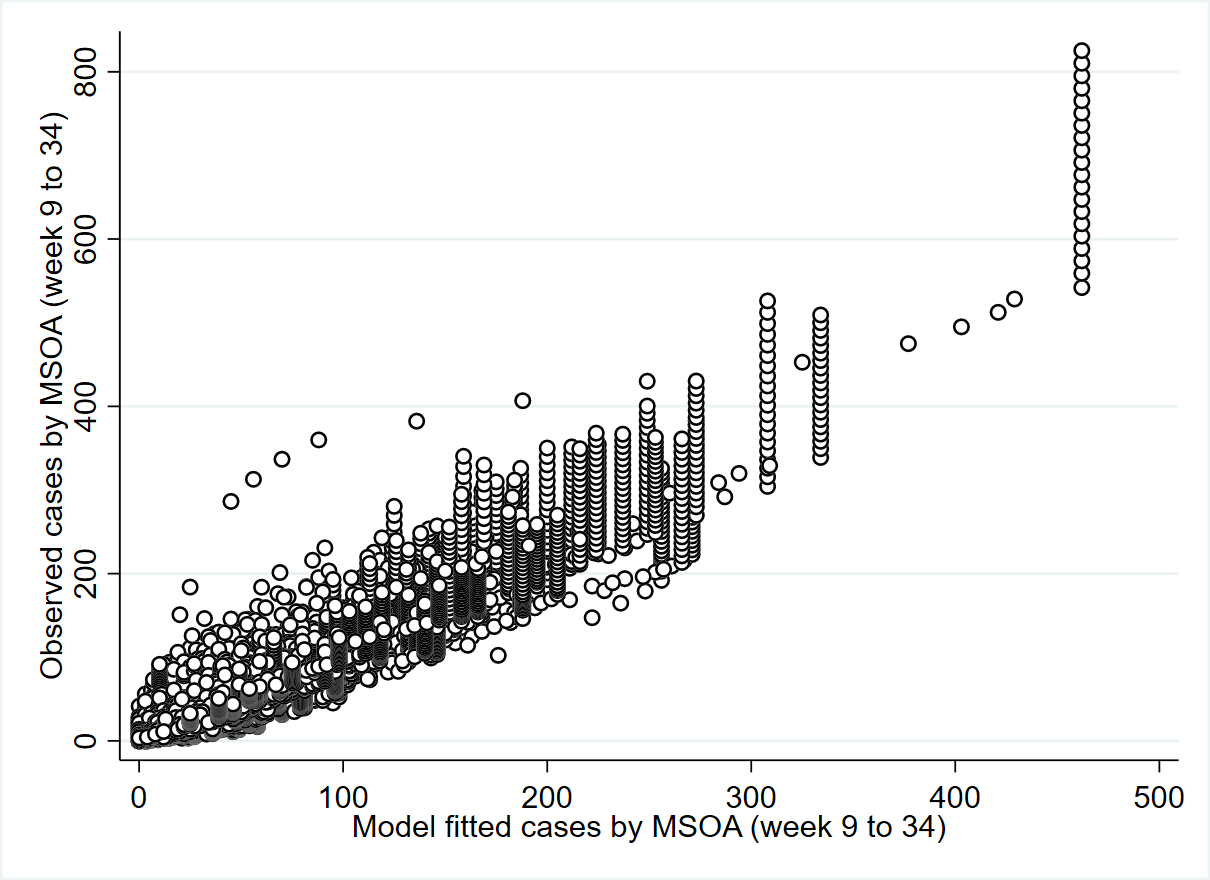


**Figure 3.1.** Scatter plot of observed cases versus model fitted cases for week 9 to week 34 of 2020.

******

**Figure 3.2.** Scatter plot of observed cases versus model fitted cases for the 20% withheld sample for week 9 to week 34 of 2020.

**Supplementary 4:** Poisson multivariable regression (non space-time, non SEIR) results for case component run in Stata 16 using observed daily reported cases by MSOA from 1 March to 18 April 2020 with population offset.

Number of obs = 162,984; (*Std. Err. adjusted for 6791 clusters in MSOA)

| **Compartment** | **Covariate** | **Coef. (β)** | **Robust Std. Err. *** | **95% CI for β** | | | **p-value** |
| --- | --- | --- | --- | --- | --- | --- | --- |
| Cases ^i^ | Cumulative mobility loss ^TV^ | +1.433 | 0.264 | +0.914 | +1.951 | <0.001 | |
|  | ln (population density) ^nTV^ | +0.099 | 0.026 | +0.048 | +0.150 | <0.001 | |
|  | Cumulative mobility loss ^TV^ x ln (population density) ^nTV^ | +0.113 | 0.035 | +0.044 | +0.181 | 0.001 | |
|  | Constant ^nTV^ | -9.371 | 0.195 | -9.754 | -8.989 | <0.001 | |
|  | ln(Population 2020) | 1 | (exposure) |  |  |  | |
| Deaths ^ii^ | Proportion of population aged 70 years or older ^nTV^ | +3.673 | 0.225 | +3.232 | +4.115 | <0.001 | |
|  | Elderly population proportion living in deprivation ^nTV^ | +1.709 | 0.113 | +1.488 | +1.930 | <0.001 | |
|  | Constant ^nTV^ | -11.101 | 0.049 | -11.200 | -11.006 | <0.001 | |
|  | ln(Population 2020) | 1 | (exposure) |  |  |  | |

TV: Time varying; nTV: not time varying; i: Variances and covariances of unstructured random effect (MSOA): .39026343 (.00471932); ii: Variances and covariances of unstructured random effect (MSOA): .55440246 (.0134037)
